# An Evaluation of a Community Hospital’s Emergency Department Ultrasonography Processes for the Diagnosis of Acute Pediatric Appendicitis

**DOI:** 10.51894/001c.11639

**Published:** 2020-01-30

**Authors:** Tanner Davis, Samuel J. Wisniewski, Heidi Suidinski, Joe Betcher

**Affiliations:** 1 Emergency Medicine Mercy Health Muskegon; 2 College of Osteopathic Medicine Statewide Campus System Michigan State University; 3 Emergency Medicine Mercy Health Muskengon

**Keywords:** quality improvement, ultrasonography, acute pediatric appendicitis

## Abstract

**CONTEXT:**

Since the 1980s, the use of ultrasonography for suspected acute pediatric appendicitis has become increasingly common. Multiple studies have suggested that ultrasound of the appendix has consistently high sensitivity and specificity when the appendix can be clearly visualized. The authors’ primary objective for this study was to retrospectively evaluate their community-based healthcare system’s processes for detecting acute pediatric appendicitis using ultrasonography.

**METHODS:**

This was a retrospective medical chart review study of data over a five-year 2014-2018 period at Mercy Health Muskegon in Muskegon, Michigan. All patients aged 3-18 years who had received an appendix ultrasound during this period were identified using the McKesson Radiology (MS) PACS-Lite computer program. Pediatric appendix ultrasound cases were collected and analyzed for sensitivity, specificity, positive predictive value, negative predictive value with 95% confidence intervals. Acute appendicitis cases had been confirmed based on pathology reports. Secondary measures including white blood cell, body mass index, and body temperature were also included in analyses.

**RESULTS:**

In this sample, the overall sensitivity at detecting acute pediatric appendicitis using ultrasonography was relatively low at approximately 42% (95% CI: 21.1 - 66.0%). On the other hand, sample specificity was quite high at 97% (95% CI: 89.9 – 99.5%). The overall positive predictive value (PPV) was 80% (95% CI: 44.2-96.5%) and the negative predictive Value (NPV) was 86% (95% CI: 75.7-92.4%). The occurrence for false positives was 20% (95% CI: 3.5-55.8%). False negatives were 14% (95% CI: 7.6-24.3%).

**CONCLUSIONS:**

The use of ultrasonography at the authors’ institution less often accurately identified cases of later-confirmed pediatric appendicitis compared to some earlier published studies. The authors concluded that this could be due to seeing a lower number of more complex/ambiguous cases of pediatric appendicitis or lack of hospital personnel’s pediatric-specific training and/or experience compared to specialty children’s hospitals. It is possible that imaging improvements could be achieved by either or a combination of: offering training sessions for general ultrasound technicians, offering training session for radiologists, and visiting pediatric physicians and ultrasound technicians. A valuable follow-up study would be to track anticipated improvements and lead to formulation of an acute pediatric appendicitis care protocol within the authors’ healthcare system.

## INTRODUCTION

Since the 1980s, the use of ultrasonography technologies for suspected acute pediatric appendicitis has been increasingly used. In 1986, the use of ultrasonography of the appendix to diagnose appendicitis was first described by Puylaert.^1^ Other studies have since suggested that ultrasound testing has consistently high sensitivity and specificity when the appendix can be clearly visualized.^2^

Ultrasounds impose lower radiation risks compared with computerized tomography (CT) or magnetic resonance imaging (MRI), and has a lower cost.^2^ In their 2014 meta-analysis, Parker et. al. examined the specific costs and relative radiation risks for ultrasound versus CT during appendicitis work-ups. This group projected that the total U.S. population cost savings in using ultrasound instead of CT as the initial imaging modality for appendicitis could save about $24.9 million annually.^2^

Parker et. al., also concluded that using ultrasound versus CT as the initial imaging modality for appendicitis would help avert 180 excess cancer deaths, with the value of the life years lost costing about $339.5 million.^2^  For these reasons, countries and organizations such as the Netherlands and the Dutch College of Surgeons now recommend that appendectomy not be carried out without prior imaging and that ultrasonography should be the first imaging modality for pediatric patients.^3^

It is theoretically easier to accurately identify an appendix with ultrasonography in most pediatric patients compared to adults due to their generally lower body mass index.  However, there have been several studies reporting relatively high accuracy with ultrasonography for appendicitis in adults as well. For example, Giliaca et. al. published a 2017 meta-analysis with sensitivity of 69% (95% CI 59-78%) and specificity of 81% (95% CI 73-88%).^4^

In 2013, Mittal et. al. performed a prospective observational study on 2,625 children aged 3 to 18 years with acute abdominal pain concerning for appendicitis.  Overall sensitivities and specificities were 72.5% (95% CI = 58.8-86.3%) and 97.0% (95% CI = 96.2-97.9%).^5^ However, sensitivity did vary depending on how frequently each hospital used this diagnostic test.  Sensitivity was 77.7% at sites in which it was used in 90% of cases, 51.6% at a site that used it in 50% of cases, and 35% at sites that used it in only 9% of cases.^5^

Fields et. al. performed a meta-analysis of point-of-care transabdominal ultrasonography performed by non-radiologist physicians for the diagnosis of acute appendicitis from 1980 to 2015. The overall sensitivity and specificity levels were 91% (95% CI = 83%-96%) and 97% (95% CI = 91%-99%); the respective positive predicative values (PPV) and negative predictive values (NPV) were 91 and 94%.^6^

Despite the high sensitivity rates demonstrated in these earlier studies, there have been other studies demonstrating lower rates for correctly identifying cases of pediatric appendicitis.  For example, Trout et. al. found in 2012 that the appendix was identified in 246 (24.4%) of 1,009 sample cases, although pediatric sonographers were significantly better at identifying the appendix compared to non-pediatric sonographers.^7^ 

Although the accuracy of readings can be quite variable, Zhang et al also reviewed about 30 articles concerning the topic and concluded that ultrasonography, CT, and MRI each had quite high diagnostic accuracy for suspected cases of pediatric appendicitis. Zhang reported the following sensitivities for each imaging modality: ultrasound= 89% (95% CI: 0.87-1.00), CT= 95% (95% CI: 0.92-0.97), MRI= 98% (95% CI: 0.96-0.99).^8^

Pediatric appendicitis care protocols are specific diagnostic algorithms designed to help clinicians determine which imaging or testing modality to start with based on patients’ clinical presentations.^9^ In recent times, more children’s hospitals have implemented pediatric appendicitis evaluation protocols, and these have been generally shown to be both safe and cost effective in general hospital settings.^9^

In 2016, Glass et. al. demonstrated that children initially evaluated for suspected appendicitis at referring hospitals were much more likely to receive a diagnostic CT and that those imaged with CT were much less likely to receive an ultrasound as their initial diagnostic test. In fact, the overall odds of receiving a CT scan was 10.9 times greater (95% CI: 9.4-12.5) at referring hospitals compared to specialty hospitals, with the odds of receiving any ultrasound at the specialty hospital 6.25 times greater (95% CI: 5.26-7.14) compared to the referral hospitals.^10^

The Pediatric Appendicitis Score (PAS) is one tool for clinicians to gauge patients’ overall clinical risk for appendicitis. The method was originally evaluated in a 2002 prospective cohort study of 1,170 patients ages 4 to 15 years with abdominal pain and has since been validated through multicenter studies.^12^The PAS observes a 10-point scoring system derived from eight variables and recommends surgical consult versus imaging (ultrasound or MRI) for equivocal scores between 4 and 6.

### Purpose of Study

The authors wished to examine how accurately their institution had used ultrasonography to detect cases of pediatric acute appendicitis.  Accuracy was gauged by measuring the sensitivity, specificity, PPV and NPV of ultrasonography as a first imaging modality in the diagnosis of pediatric acute appendicitis.

The authors hoped to gain insights as to how their community-based hospital compared to diagnostic accuracy levels previously reported in the literature. The authors hoped to identify improvement areas for their institution's use of ultrasonography testing, which could potentially reduce both radiation risks for pediatric patients and medical costs.  Results could also serve as a starting point for the authors to implement their own pediatric appendicitis protocol or to consistently apply a clinical tool such as the PAS when deciding between various imaging modalities.

## METHODS

The authors extracted retrospective electronic health record data from a five-year period defined as (1/1/2014-10/31/2018) in their community hospital.  The Institutional Review Board had approved the research project before any form of data were collected. Subject selection included children aged 3-18 years old who presented to one of three Mercy Health Muskegon facilities (Hackley campus, Mercy campus, Mercy Health Pavilion) with acute abdominal pain suggesting acute appendicitis. 

Children suspected of having appendicitis had subsequently received a limited transabdominal ultrasound performed by a trained ultrasound technician in the radiology department. None of the ultrasounds reviewed in this study included point-of-care bedside ultrasounds performed by medical providers. Patients were excluded if they had already received an appendectomy. Patients were evaluated for inclusion/exclusion criteria by searching several databases. 

First, the McKesson Radiology (MS) PACS-Lite computer program was used to search for patients who were aged 3-18 years on whom a limited abdominal ultrasound of the right lower quadrant (RLQ) had been completed during the study period. A secure, password protected data spreadsheet was also used to store patient data from the hospital’s electronic health record including each patient’s gender, age, BMI, maximum body temperature at time of ultrasound, and serum WBC.

The authors also recorded each case’s “final ultrasound impression” as interpreted by a radiologist, “final clinical diagnosis” as documented by each medical provider who evaluated the patient, and each “pathology report” as documented by a pathologist if the patient subsequently underwent appendectomy.

The authors had initially estimated that collecting data from at least 50 cases would provide them with an adequate level of statistical power and therefore 95% confidence intervals. This sample size has been quoted in other recent pediatric ultrasound papers.^11^

### Analytic Methods 

Sensitivity, specificity, PPV and NPV estimates were calculated by the second author (SJW).  Data were also assessed with 95% confidence intervals. Data concerning secondary measures (i.e., increased BMI, white blood cell count (WBC) and body temperature) that may have improved or impeded the process of detecting cases of pediatric appendicitis were also collected. Statistical analyses were performed using SPSS Version 25.

## RESULTS

There were a total of N = 88 pediatric patients from whom largely-complete data were extracted. Approximately 30 (34.1%) sample patients were male and 58 (65.9%) were female. Their average (mean) age was 10.1 years (SD = 4.39), average BMI was 19.34 (SD = 4.62), average body temperature in degrees Fahrenheit was 99.24 (SD = 1.55), and their WBC averaged 11.28 (SD = 5.00). (Table 1)

**Table 1:** Sample Characteristics (N = 88)

**Gender**	
* Male*	30 (34.1%)
* Female*	58 (65.9%)
**Temperature F**	99.24 (SD = 1.55)
**Age** (mean)	10.1 (SD = 4.39)
**BMI** (mean)	19.34 (SD = 4.62)
**WBC** (mean, N = 83)	11.28 (SD 5.00)

Data concerning primary outcomes were obtained from all 88 subjects, and the occurrence of appendicitis was noted as approximately 10 (11.4%) on Ultrasound Final Impression, 19 (21.6%) on Final Clinical Diagnosis, and 18 (20.5%) on the Pathology Report. (Table 2)

**Table 2:** Frequencies of Ultrasound, Clinical Diagnosis and Pathology Findings

**Ultrasound Final Impression**	**N = 88**
*Appendicitis*	10 (11.4%)
NVOA*	78 (88.6%)
**Final Clinical Diagnosis**	
*Appendicitis*	19 (21.6%)
*Other Diagnosis*	69 (78.4%)
**Pathology Report**	
*Pathology *	18 (20.5%)
*No Pathology*	69 (78.4%)

*NVOA = Non-visualization of Appendix

In addition, the mean of the three selected secondary measures (i.e., BMI, WBC, and body temperature) were calculated to compare the Ultrasound Final Impression subgroups of appendicitis and Nonvisualization of the Appendix (NVOA). The Ultrasound Final Impression: Appendicitis subgroup had average BMI = 19.41 (SD 5.31), average WBC = 16.78 (SD 5.42), and average temperature in degrees Fahrenheit = 100.22 (SD 1.54). The Ultrasound Final Impression: NVOA subgroup had average BMI = 19.32 (SD 4.44), average WBC = 9.62 (SD 3.51), and average body temperature = 98.97 (SD 1.45). (Table 3)

**Table 3:** Mean BMI, WBC, and Body Temperature by Ultrasound Final Impression

	**Appendicitis**	NVOA *
**BMI**	19.41 (SD = 5.31)	19.32 (SD = 4.44)
**WBC**	16.78 (SD = 5.42)	9.62 (SD = 3.51)
**Temp**	100.22 (SD = 1.54)	98.97 (SD = 1.45)

*NVOA = Non-visualization of Appendix

## Sensitivity and Specificity

The authors also examined the validity values for sensitivity and specificity, and the reliability values for true positives and true negatives. The sensitivity, or the ability of the test to correctly identify an individual as “diseased” of the Ultrasound Final Impression in accurately recognizing appendicitis verified on Final Clinical Diagnosis was low, at approximately 42% (95% CI: 21.1 - 66.0%).

The specificity on the other hand, or the ability of the test to correctly identify an individual as disease-free, was high, with the accuracy of a non-visualized appendix from Ultrasound Final Impression in predicting a lack of appendicitis on Final Clinical Diagnosis at 97% (95% CI: 89.9 – 99.5%).

### Predictive Values

The number of True Positives (i.e., PPV), which measures the percentage of patients with a positive test results who actually have appendicitis, was approximately 80% (95% CI: 44.2-96.5%). Similarly, the number of True Negatives (NPV) which measures the percentage of patients with a negative test who do not have appendicitis, was 86% (95% CI: 75.7-92.4%). False Positives, tests which labeled individual as diseased when they were not, occurred 20% (95% CI: 3.5-55.8%).  False Negatives, when a test has wrongly labeled a diseased person as “normal”, 14% (95% CI: 7.6-24.3%). (Table 4)

**Table 4:** Reliability and Validity of Diagnoses

**Sensitivity:** 0.42 (95% CI: 0.211-0.660)
**Specificity:** 0.97 (95% CI: 0.899-0.995)
**True Positive (PPV):** 0.8 (95% CI: 0.442-0.965)
**True Negative (NPV):** 0.86 (95% CI: 0.757-0.924)
**False Positive:** 0.20 (95% CI: 0.035-0.558)
**False Negative:** 0.14 (95% CI: 0.076-0.243)

## DISCUSSION

We had initially expected to find lower sensitivities and specificity rates at our institution when evaluating for pediatric acute appendicitis using ultrasonography.  However, it was still surprising to find our sensitivity to be as low as 42%. Our system’s rate of NVOA was also quite high at 88.6%.  However, there could still be value derived from having such a high NVOA as our negative predictive value was 86%. This finding certainly represents an area for improvement, as there were also quite a few cases with follow up CT imaging which may not have been required due to the NVOA read on ultrasound.

In 2014, Nah et. al. found that the likelihood of appendicitis in children was less than 2% with NVOA and no evidence of secondary inflammatory changes on ultrasound.^13^ Nikolaidis et. al. also reported a NVOA rate of 13% for 366 adults, and only one of 46 (12.6%) NVOA cases was verified as acute appendicitis.^14^ Although it may be more reassuring to rule out appendicitis by visualizing a normal appendix, NVOA may still possess a high enough negative predictive value to prevent clinicians from ordering subsequent CT films.

After reviewing our results, it is possible that the reason our US tests less frequently identified later confirmed cases of acute appendicitis may be due to our clinicians having had less frequent exposure to potential pediatric appendicitis cases, and fewer on-site ultrasound technician trainings compared to pediatric-trained ultrasonographers in pediatric specialty hospitals.  Our radiologists may also have been less familiar reading more complex/ambiguous pediatric appendix ultrasound studies compared to radiologists at pediatric hospitals.

There have been relatively few published studies comparing community-based hospitals to academic pediatric specialty hospitals. However, two earlier-cited studies demonstrated rates that were fairly similar to ours: Mittal et. al. with an overall sensitivity for acute appendicitis was only 35% at sites using ultrasound in only 9% of cases^4^ and Trout et. al. reporting a rate of only 24.4% of pediatric cases where the appendix was identified.^7^

Due to the low appendix detection rates at Mercy Healthy Muskegon, we also found that 33 (37.5%) of our 88 sample patients received possibly unnecessary follow-up CT scan on the same day during which their appendix ultrasound had been performed.  It was also concerning that our False Negative rate was 14% since these represent confirmed appendicitis cases which potentially could have been detected by ultrasound alone, thus preventing any follow-up CT films. 

In our sample, there were four NVOA cases that were particularly concerning. Similar to earlier studies,^13^ the appendix in each of these cases had not been visualized on ultrasound but later revealed secondary features of acute appendicitis (e.g., an appendicolith within the appendix lumen X2, dilated appendix with periappendiceal stranding X2) on follow-up CTs. Three additional sample ultrasound studies were later read by outside radiologists in referral pediatric hospitals and deemed to be inadequate studies when accepting transferred patients.

In regard to our selected secondary measures, the average (mean) WBC and body temperatures were higher in the confirmed appendicitis sample subgroup (WBC = 16.78; body temperature 100.22) compared to the NVOA group (WBC = 9.62; body temperature = 98.97). This could be expected that appendicitis would likely have a higher WBC and temperature compared to a final diagnosis not caused by bacterial/infectious etiology. However, of the 18 cases of acute appendicitis later confirmed by pathology, only 10 (55.5%) had been initially identified by ultrasound. In effect, if the eight NVOA cases had been accurately identified as acute appendicitis on ultrasound, it is possible that the difference between each subgroup’s average WBC and temperature could have been even larger.

Interestingly, the average BMI was only slightly higher in the appendicitis subgroup (19.41, SD = 5.31) compared to the NVOA group (19.32, SD = 5.44). The authors expected the appendicitis cases identified on ultrasound would have a smaller average BMI than the NVOA cases since it is theoretically easier to identify the appendix on lower BMI patients possessing less abdominal fat.

## CONCLUSIONS

Overall, our institutional rate of accurately identifying pediatric appendicitis by ultrasonography was lower than anticipated, with a lower sensitivity than earlier published studies. This is most likely related to our hospital personnel’s less frequent exposure to more complex or ambiguous cases of pediatric appendicitis.

We have also concluded that our specificity rates could be improved by further training our ultrasound technicians and evaluating later incomplete studies with our radiologists. As we exceeded our estimated sample size goal of 50 patients with a sample size of N = 88, it is quite likely that these results accurately represent the pediatric ultrasound for acute appendicitis patterns at similar community-based health systems. 

In many settings, it may be beneficial to have collaborative training sessions involving hospital’s sonographers, radiologists, ultrasound-trained physicians, and visiting pediatric trained/experienced sonographers.  Ideally, improving ultrasonography work-up patterns for pediatric patients could potentially reduce unnecessary radiation exposure risks and medical costs related to follow up CT imaging. Further larger-scale studies are also required to evaluate the impact of ultrasonography training sessions for pediatric acute appendicitis protocols at similar community-based hospitals.   

NOTE: The review of this paper was coordinated by SMRJ Chief Editor William Corser

### Conflict of Interest

The authors declare no conflict of interest.
